# Bullying victimization and suicide attempts among adolescents in 41 low- and middle-income countries: Roles of sleep deprivation and body mass

**DOI:** 10.3389/fpubh.2023.1064731

**Published:** 2023-02-22

**Authors:** Wenxin Bao, Yi Qian, Wenjing Fei, Shun Tian, Yiran Geng, Shaishai Wang, Chen-Wei Pan, Chun-Hua Zhao, Tianyang Zhang

**Affiliations:** ^1^School of Public Health, Medical College of Soochow University, Suzhou, China; ^2^Mental Health Center, West China Hospital, Sichuan University, Chengdu, China; ^3^Department of Obstetrics and Gynecology, The First Affiliated Hospital of Soochow University, Suzhou, China; ^4^Infectious Disease Control Division, Xiangcheng District Center for Disease Control and Prevention, Suzhou, China; ^5^Department of General Medicine, Big Data Center, The Affiliated Suzhou Hospital of Nanjing Medical University, Suzhou Municipal Hospital, Nanjing Medical University, Suzhou, China

**Keywords:** bullying victimization, suicide attempts, sleep deprivation, body mass index, adolescents

## Abstract

**Background:**

Suicide is the fourth leading cause of death for adolescents, and globally, over 75% of completed suicides occur in low- and middle-income countries (LMICs). Bullying has been proven to be closely related to suicide attempts. However, further understanding of the mechanisms underlying the relationship between bullying and adolescents' suicide attempts is urgently needed.

**Methods:**

We used data from the Global School-based Student Health Survey (GSHS) (2010–2017) from 41 LMICs or regions. This study was based on questions assessing bullying victimization, suicide attempts, sleep deprivation, and body mass. Chi-square tests were used to explore the correlations among the main variables. The mediating role of sleep deprivation and the moderating role of body mass index (BMI) were analyzed using PROCESS.

**Results:**

The results showed a positive association between bullying victimization and suicide attempts. Sleep deprivation partially mediated the relationship between the frequency of being bullied and suicide attempts. In addition, sleep deprivation played a full or partial mediating role in the relationship between different types of bullying and suicide attempts. BMI moderated the relationships between the frequency of being bullied and suicide attempts, between being made fun of about one's body and sleep deprivation, and between sleep deprivation and suicide attempts.

**Conclusion:**

Being bullied has a positive effect on suicide attempts, which is mediated by sleep deprivation and moderated by body mass. The results of this study are consistent with the stress-diathesis model of suicide, suggesting that being bullied is one of the stressors of suicide in adolescents, while sleep deprivation and body mass are susceptibility diatheses of suicide. The results are conducive to identifying adolescents at a high risk of suicide, suggesting that there is a need to pay more attention to bullied adolescents, especially their sleep quality and body mass, and design effective intervention measures to improve the current situation of adolescent suicide in LMICs.

## Background

Suicide is the fourth leading cause of death for adolescents, and it has become a major public health concern (1). Rates of attempted suicide and death by suicide have been increasing for more than a decade (2). The global age-standardized suicide rate was 9.0 per 100,000 population for 2019, while the majority of deaths by suicide occurred in low- and middle-income countries (LMICs), with a 77% probability. It is worth noting that 88% of adolescents who died by suicide were from LMICs, where nearly 90% of the world's adolescents live (1). According to research, suicide and bullying are strongly linked (3). Bullying is a type of aggressive behavior that occurs repeatedly in interpersonal relationships where power imbalances exist, increasing the risk of physical and psychosocial problems through its influence (4). The involvement of adolescents in bullying is becoming an important public health problem that is attracting the attention of researchers.

### Suicide attempt

Suicide can be classified as suicidal ideation, suicidal plan, and suicide attempt. In a previous study, suicidal ideation was reported by 24.66%, suicidal planning was reported by 15.55%, and suicide attempts were reported by 4.37% of adolescents (5). Suicide attempts are the most serious of the three, directly damaging health, with the most serious consequences being disability or even death. Attempted suicide refers to an intentional, self-inflicted, life-threatening act that results in physical injury but not death (6). Suicide attempts are up to 30 times more common than suicides; however, they are important predictors of repeated attempts and completed suicides (7). Suicide attempts are undertaken most frequently by young people (8). Therefore, we need to pay more attention to suicide among teenagers, especially in LMICs.

In 1960, Bleuler highlighted the role of diathesis-stress interactions in the course of schizophrenia (9), and in 1980, DeCatanzaro extended the concept to the mechanism of suicide (10). In the stress-diathesis model of suicide, stress is considered to be the stressful environment or life events (such as tension in relationships and academic pressure) that trigger suicidal behavior, while diathesis refers to the individual vulnerability or susceptibility to suicide. The stress-diathesis model holds that suicide is the result of an interaction between state-dependent (environmental) stressors and a similarly characterized diathesis or susceptibility to suicidal behavior. It was believed that diathesis was primarily a biological trait produced by genetic predisposition, that is, genetic vulnerability (11, 12). Studies in recent years have also expanded the term to include cognitive and social factors that predispose people to suicidal behavior, such as traits of impulsivity (13). Among the stressors that trigger suicidal behavior in adolescents, we need to pay special attention to bullying. Bullying is a deliberate and aggressive behavior commonly seen in school settings. The main social living environment of adolescents is school, and the adverse events occurring in school easily affect the mental health of adolescents. Researchers have found that being a victim of bullying is significantly associated with reports of suicidal ideation and attempts (14–17). In summary, being bullied can be considered a significant stressor in adolescent suicide in LMICs.

### The impact of bullying victimization on suicidal behavior

Bullying is an intentional and aggressive behavior that is performed repeatedly and based on an imbalance of power between the perpetrator and victim (18, 19). Our research is based on the Global School-based Student Health Survey (GSHS) initiated by the WHO, which uses a self-administered questionnaire to obtain data concerning young people's health behavior and protective factors related to the leading causes of morbidity and mortality among children and adults worldwide. The GSHS subdivides the content of bullying into being hit, kicked, pushed, shoved, or locked indoors; being made fun of because of race, nationality, or color; being made fun of because of religion; being made fun of with sexual jokes, comments, or gestures; being left out of activities on purpose or being completely ignored; being made fun of because of how one's body or face looks; and being bullied in some other way. Previous research has shown that male subjects appear to engage in more physical forms and female subjects appear to engage in more relational forms of bullying and that physical bullying is more prevalent in younger age groups (20–22). In the GHSH's detailed division of bullying, being hit, kicked, pushed, shoved, and locked indoors are types of physical bullying, and the other types are more prone to relational bullying. In addition, former studies have found that bullying is related to prejudice and that the risk of bullying and victimization is not equal across student groups. A number of studies have indicated that students with disabilities or suffering from obesity and those belonging to ethnic or sexual minorities are at greater risk of being victimized than their peers (23–26). This suggests that the occurrence of bullying behaviors is also related to some inherent characteristics of the bullied. Bullying has negative health consequences for both bullies and victims, and it can have a negative impact on bystanders as well. Several longitudinal studies from different countries, along with systematic reviews and meta-analyses, have demonstrated the relationship between school bullying or the experience of being victimized and later health outcomes (27–30). These associations hold even after controlling for other childhood risk factors (31). Bullying is even a major risk factor for adolescent suicide, which has been shown in some longitudinal and cross-sectional studies (32–35). Physical bullying perpetration, in particular, may put adolescents in situations where they are actually injured (i.e., victim defends themselves) or where there is a threat of injury. Thus, bullies may repeatedly experience physical pain and threatening situations. Through habituation and conditioning, exposure to these types of painful and provocative events makes people more capable of making potentially lethal suicide attempts (36). Research by Barzilay et al. also showed that physical victimization is related to suicidal ideation and that relationship victimization is related to suicide attempts (37). In addition, extensive research evidence indicates that bullying victimization is a factor related to sleep impairment in adolescents.

### The mediating effect of sleep deprivation

The average sleep needed for an adolescent to maintain health is 8–10 h per night (38). The Youth Risk Behaviour Survey found that 72.7% of students reported an average of <8 h of sleep on school nights (39). As many as one-fourth of adolescents report sleeping 6 h or less per night (40). Therefore, sleep deprivation is a significant public health concern (41). The effects of sleep deprivation on adolescents can be particularly detrimental, impacting their ability to learn, manage mood and anxiety, develop relationships, avoid accidents, and stay physically healthy (42). However, sleep deprivation, but not eveningness, is significantly linked to suicidality after controlling for mood symptoms (43). A number of recent studies have identified sleep deprivation as a modifiable, independent suicide risk factor (44). Studies have shown that improved sleep might reduce suicide risk (45). In conclusion, sleep deprivation is one of the possible diatheses in the stress-diathesis model of suicide.

Adolescence is a period marked by changes in sleep timing and patterns, and several factors may contribute to sleep deprivation in adolescents, including biological and psychological aspects. Physiologically, the adolescent sleep-wake cycle is shifted, and melatonin secretion is delayed, leading to a delay in the circadian rhythm, resulting in a mismatch between adolescents' preferred sleep time and social needs such as going to school. From a psychological point of view, stressful life events such as academic pressure and interpersonal relationships, as well as mental health problems such as depression, will all lead to sleep loss over worry (SLOW) in adolescents. Bullying victimization is also an important factor in this. Previous research indicated that bully victimization was consistently and positively linked to SLOW, with greater odds of sleep deprivation among students with severe SLOW who were bullied for 3 days or more (46). A similar pattern was found across all bullying roles, with more sleep disturbances for victims and bully victims (47). It is possible that the fear associated with being bullied and rumination over bullying experiences may interfere with the onset of sleep and contribute to poor sleep quality. Evidence supports a robust association between bullying victimization and sleep problems (i.e., difficulties falling or remaining asleep). Bullying victimization is thus an independent risk factor for sleep disturbances and warrants deeper investigation, particularly in adolescence. Based on the above, we find that sleep deprivation is related to bullying and suicide. The purpose of our study is to explore whether sleep deprivation plays a mediating role in the pathway from bullying to suicide.

### Body mass as a moderator

As early as 1966, increasingly well-powered and well-designed studies have demonstrated a relationship between a heavier BMI and a lower risk of suicide (48). A systematic review and meta-analysis conducted by Perera and colleagues showed a negative correlation between BMI and completed suicide; the evidence for the association between BMI and suicide attempt was inconsistent, and when confounding factors were considered, there was no correlation between BMI and suicidal ideation (49). However, there have also been different research results on the relationship between body mass and suicide. Dong and colleagues found that both lower and higher BMI were associated with an elevated risk for attempted suicide and that extreme obesity (BMI ≥ 40 kg/m^2^) was significantly associated with attempted suicide (50). We think BMI might also be a vulnerability trait of suicide. However, there is still controversy about the relationship between body mass and suicidal behavior, which urgently needs to be explored, as was done in this study.

The relationships between bullying victimization, sleep deprivation and suicide are not always the same in all cases. Adolescents with overweight or obese have significantly greater odds than their healthy-weight peers of being victims of bullying (25). Previous findings indicated that 64% of the study participants reported weight-based victimization at school and that the risk of weight-based victimization increased with body weight (51). However, research by Thakkar et al. in India found that higher BMI corresponded to less victimization for boys (52). Koyanagi et al. found that compared with normal weight, being overweight and obese were associated with significantly higher odds of any form of bullying victimization only among girls. Among boys, these associations were not significant overall (53).

Studies have shown that a reduction in sleep time is related to an increase in eating (54). Short sleep times reduce the activity of circuits related to control and inhibition, leading to poor resistance to temptation and self-control in the presence of food, that is, increasing the level of external eating, which may lead to an increase in BMI (55). In addition, from a medical point of view, people with high BMI are susceptible to obstructive sleep apnea (OSA), which can lead to poor sleep quality (48). In summary, we deduced a vicious cycle between obesity and lack of sleep. Obese people are prone to lack of sleep, and poor sleep can also aggravate the occurrence of obesity. Based on previous literature, we have known that sleep deprivation has a positive effect on suicide (44, 45). Therefore, it is not difficult to infer that body mass can further increase the probability of bullying victims engaging in suicidal behavior through a positive effect on sleep deprivation. Based on the theoretical and empirical results discussed earlier, body mass may play a moderating role in the relationship between bullying victimization, sleep deprivation, and suicidal behavior.

### This study

The majority of previous research has focused on the association between bullying victimization and suicide. However, studies clarifying the interaction among bullying victimization, sleep deprivation, and suicide are scarce, and few studies have discussed the role of body mass. In addition, most of the previous studies have focused only on the impact of the frequency of being bullied on suicide, while this study examined both the frequency of being bullied and the different types of bullying. There is no clear understanding of the underlying mediating mechanisms and moderating factors that may affect this association. In particular, this study had multiple aims. First, we examined whether bullying victimization has an effect on the suicide attempt of adolescents. Second, we examined whether sleep deprivation mediates the relationship between bullying victimization and suicide attempts among adolescents. Third, we tested whether the relationship between bullying victimization and suicide attempts through sleep deprivation is regulated by body mass. In summary, our hypotheses were as follows:

Hypothesis 1. Being bullied is positively associated with suicide attempts in adolescents, and this applies to different types of bullying content.

Hypothesis 2. Being bullied predicts suicide attempts in adolescents through sleep deprivation. Being bullied can cause the victim's sleep condition to deteriorate, thereby further inducing suicide attempts.

Hypothesis 3. BMI moderates the relationship between bullying victimization and suicide attempts in adolescents through sleep deprivation. Specifically, the influence of being bullied on sleep deprivation and the influence of sleep deprivation on suicide attempts of adolescents differ in different BMI groups, and this effect is more significant in people with high BMI.

## Methods

### Sources of data

We analyzed the most recent Global School-based Student Health Survey (GSHS) data (2010–2017) from 41 LMICs or regions. GSHS data, methods, and main findings are available on the WHO (http://www.who.int/ncds/surveillance/gshs/en/) and the Centers for Disease Control and Prevention (CDC)'s websites. According to the World Health Organization (WHO), adolescents are individuals aged between 10 and 19 years, and the GSHS is a collaborative surveillance project designed to help countries measure and assess behavioral risk factors and protective factors in 10 key areas among young people mostly aged 12–17 years (53, 56). To facilitate comparisons between countries, we excluded survey responses collected before 2010. Only the most recent GSHS was analyzed for countries that had completed more than one survey. Our final sample size is 121,869.

GSHS consistently used a two-stage cluster sampling strategy to obtain a nationally representative sample of middle school students in all countries. The first stage involved selecting random schools from a country based on their size in proportion to the probability distribution. During the second stage, classes from the schools were selected with systematic equal probability sampling and random start. In the sampling frame, all students from the selected school classes were included. It is possible to translate the questionnaire into any language. Students' records are identified, and data were collected by automated optical character recognition on computer-scannable answer sheets.

The GSHS administration is approved by either a Ministry of Education or a Health Research Ethics Committee in each participating country. Participants and their guardians in each country provided verbal or written consent.

### Main variables

The following aspects were the focus of our analysis: situation of being bullied, suicide attempt, sleep deprivation, and BMI. These questions are available from the questionnaire of GSHS (57).

The situation of being bullied consisted of two parts: the frequency of being bullied and the types of bullying. “The frequency of being bullied” was assessed with the following question: “During the past 30 days, on how many days were you bullied?” The response options for the question were “0 days,” “1 or 2 days,” “3 to 5 days,” “6 to 9 days,” “10 to 19 days,” “20 to 29 days,” and “All 30 days.” “The types of bullying” were assessed by the following question: “During the past 30 days, how were you bullied most often?” The response options for the question were “I was not bullied during the past 30 days,” “I was hit, kicked, pushed, shoved around, or locked indoors,” “I was made fun of because of my race, nationality, or color,” “I was made fun of because of my religion,” “I was made fun of with sexual jokes, comments, or gestures,” “I was left out of activities on purpose or completely ignored,” “I was made fun of because of how my body or face looks,” and “I was bullied in some other way.”

“Suicide attempt” was assessed by the question “During the past 12 months, how many times did you actually attempt suicide.” The response options for the question were “0 times,” “1 time,” “2 or 3 times,” “4 or 5 times,” and “6 or more times,” In the chi-square test, we coded the answer “0 times” as “does not have the experience of suicide attempt” and the responses “1 time,” “2 or 3 times,” “4 or 5 times,” and “6 or more times” as “has the experience of suicide attempt.”

“Sleep deprivation” was assessed by the following question: “During the past 12 months, how often have you been so worried about something that you could not sleep at night?” The response options for the question were “Never,” “Rarely,” “Sometimes,” “Most of the time,” and “Always.”

“BMI” was calculated with data on height and weight using the formula BMI = weight (kg)/height (m) squared.

### Control variables

Age, gender, other mental health problems (loneliness), and relationships with peers (having close friends, if other students are kind and helpful or not) of the respondent were included as covariates.

### Statistical analyses

The statistical analyses were conducted in SPSS v.21.0 with the addition of the PROCESS plug-in for moderation and mediation analyses. The analysis of correlations among the main variables was carried out using chi-square tests. Then, the mediating role of sleep deprivation and the moderating role of BMI were analyzed in PROCESS, and robust standard errors and bootstrap confidence intervals were derived from 5,000 bootstrap samples. The result is statistically significant if the confidence interval excludes 0. We adjusted for covariates in the moderation and mediation analyses.

## Results

We identified 41 LMICs with the GSHS datasets, removed the cases with missing values, and finally included a sample size of 121,869. The proportion of male participants in the sample was 44.8%. The results indicated that 9.7% of the participants reported having experienced a suicide attempt, and 40.9% of the participants reported having been exposed to bullying.

### Descriptive statistics and chi-square test

The descriptive statistics for the variables and the results of the chi-square test are shown in Table 1. We found that being bullied more frequently can lead to higher rates of suicide attempts. Among the types of bullying, “make fun because of religion” was the most strongly associated with suicide attempts. In total, 25.0% of people who were made fun of because of their religion attempted suicide. A total of 79.8% of adolescents reported having sleep deprivation. When the degree of sleep deprivation was more severe, the rate of suicide attempts was higher. In total, 7.90% of the adolescents who attempted suicide had a BMI lower than 18.5, and 9.80% of them had a BMI higher than 30. Furthermore, the chi-square test of suicide attempts among groups was statistically significant.

**Table 1:** Factors associated with suicide attempts among adolescents (*n* = 121,869).

**Variables**	**Categories**	**Suicide attempts**		
		**Yes**	**No**	***p***
Age	12 years old	500 (7.30%)	6,390 (92.70%)	<0.001
	13 years old	1,892 (8.50%)	20,461 (91.50%)	
	14 years old	2,855 (10.20%)	25,205 (89.80%)	
	15 years old	2,988 (10.60%)	25,265 (89.40%)	
	16 years old	2,501 (10.80%)	20,715 (89.20%)	
	17 years old	1,064 (8.10%)	12,033 (91.90%)	
Sex	Male	4,301 (7.90%)	50,250 (92.10%)	<0.001
	Female	7,499 (11.10%)	59,819 (88.90%)	
BMI	<18.5	2,746 (7.90%)	31,986 (92.10%)	<0.001
	18.5–24.9	7,701 (10.50%)	65,820 (89.50%)	
	25–30	931 (10.00%)	8,381 (90.00%)	
	>30	422 (9.80%)	3,882 (90.20%)	
Feeling lonely	Never	2,373 (5.70%)	39,317 (94.30%)	<0.001
	Rarely	2,273 (7.50%)	27,834 (92.50%)	
	Sometimes	4,027 (10.70%)	33,558 (89.30%)	
	Most of the time	1,974 (22.10%)	6,948 (77.90%)	
	Always	1,153 (32.30%)	2,412 (67.70%)	
Close friends	0	1,083 (19.80%)	4,394 (80.20%)	<0.001
	1	1,497 (13.80%)	9,342 (86.20%)	
	2	1,875 (12.40%)	13,245 (87.60%)	
	3 or more	7,345 (8.10%)	83,088 (91.90%)	
Sleep deprivation	Never	2,384 (5.00%)	45,174 (95.00%)	<0.001
	Rarely	2,931 (8.80%)	30,354 (91.20%)	
	Sometimes	3,939 (12.50%)	27,563 (87.50%)	
	Most of the time	1,720 (24.60%)	5,283 (75.40%)	
	Always	826 (32.80%)	1,695 (67.20%)	
The frequency of being bullied	0 days	6,133 (6.90%)	82,784 (93.10%)	<0.001
	1 or 2 days	2,972 (14.40%)	17,724 (85.60%)	
	3–5 days	1,112 (19.80%)	4,517 (80.20%)	
	6–9 days	498 (21.70%)	1,796 (78.30%)	
	10–19 days	307 (20.90%)	1,162 (79.10%)	
	20–29 days	177 (26.20%)	499 (73.80%)	
	All 30 days	601 (27.50%)	1,587 (72.50%)	
Types of bullying	Not bullied	6,973 (7.30%)	88,405 (92.70%)	<0.001
	Kicked, pushed, or shoved	669 (20.60%)	2,586 (79.40%)	
	Made fun of race	533 (20.60%)	2,055 (79.40%)	
	Made fun because of religion	224 (25.00%)	673 (75.00%)	
	Made fun of about sex	898 (19.80%)	3,635 (80.20%)	
	Left out of activities	303 (18.20%)	1,361 (81.80%)	
	Made fun of about body	873 (18.00%)	3,968 (82.00%)	
	Some other way	1,327 (15.20%)	7,386 (84.80%)	

### The mediating effect of sleep deprivation

Table 2 illustrates the mediating effect of sleep deprivation. Sleep deprivation partially mediated the effect between the frequency of being bullied and suicide attempts. As expected, the total effect of the frequency of being bullied (β = 0.085, *t* = 61.999, *p* < 0.001) on suicide attempts in the absence of sleep deprivation was significant. When sleep deprivation was added to the analysis as a mediator, the effects of the frequency of being bullied on sleep deprivation (β = 0.163, *t* = 62.713, *p* < 0.001) were positive, and sleep deprivation (β = 0.086, *t* = 57.316, *p* < 0.001) still positively predicted suicide attempts. Bootstrapping indicated that sleep deprivation played a significant role in explaining the association between the frequency of being bullied and suicide attempts (indirect effect = 0.014, 95% CI = 0.013–0.015). Regarding the different types of bullying, sleep deprivation had a full mediating effect between the associations of suicide attempts with being kicked, pushed, or shoved; being made fun of about one's sex; being left out of activities; and being bullied some other way. It had a partial mediating effect on the associations between being made fun of about one's race, being made fun of about one's body, and suicide attempt. The details are shown in Table 2.

**Table 2:** The mediating effect of sleep deprivation as a dependent variable (*n* = 121,869).

	**Model 1**		**Model 2**		**Model 3**	
	**Suicide attempts**		**Sleep deprivation**		**Suicide attempts**	
**Variable**	β	***t***	β	***t***	β	***t***
The frequency of being bullied	0.085	61.999^***^	0.163	62.713^***^	0.071	51.704^***^
Sleep deprivation					0.086	57.316^***^
*R* ^2^	0.031		0.031		0.056	
*F*	3,843.840		3,932.895		3,616.259	
Kicked, pushed, or shoved	0.229	23.822^***^	0.367	20.300^***^	0.197	20.739^***^
Made fun of race	0.239	22.215^***^	0.508	25.209^***^	0.194	18.281^***^
Made fun because of religion	0.317	17.508^***^	0.577	17.015^***^	0.266	14.901^***^
Made fun of about sex	0.221	26.962^***^	0.548	35.626^***^	0.173	21.298^***^
Left out of activities	0.203	15.214^***^	0.54	21.582^***^	0.156	11.811^***^
Made fun of about body	0.204	25.651^***^	0.541	36.290^***^	0.156	19.862^***^
Some other way	0.141	23.331^***^	0.407	35.921^***^	0.105	17.585^***^
Sleep deprivation					0.087	58.051^***^
*R* ^2^	0.023		0.037		0.050	
*F*	417.521		671.44		796.665	

*** *p* < 0.001.

### Body mass as a moderator

**Figure 1:**
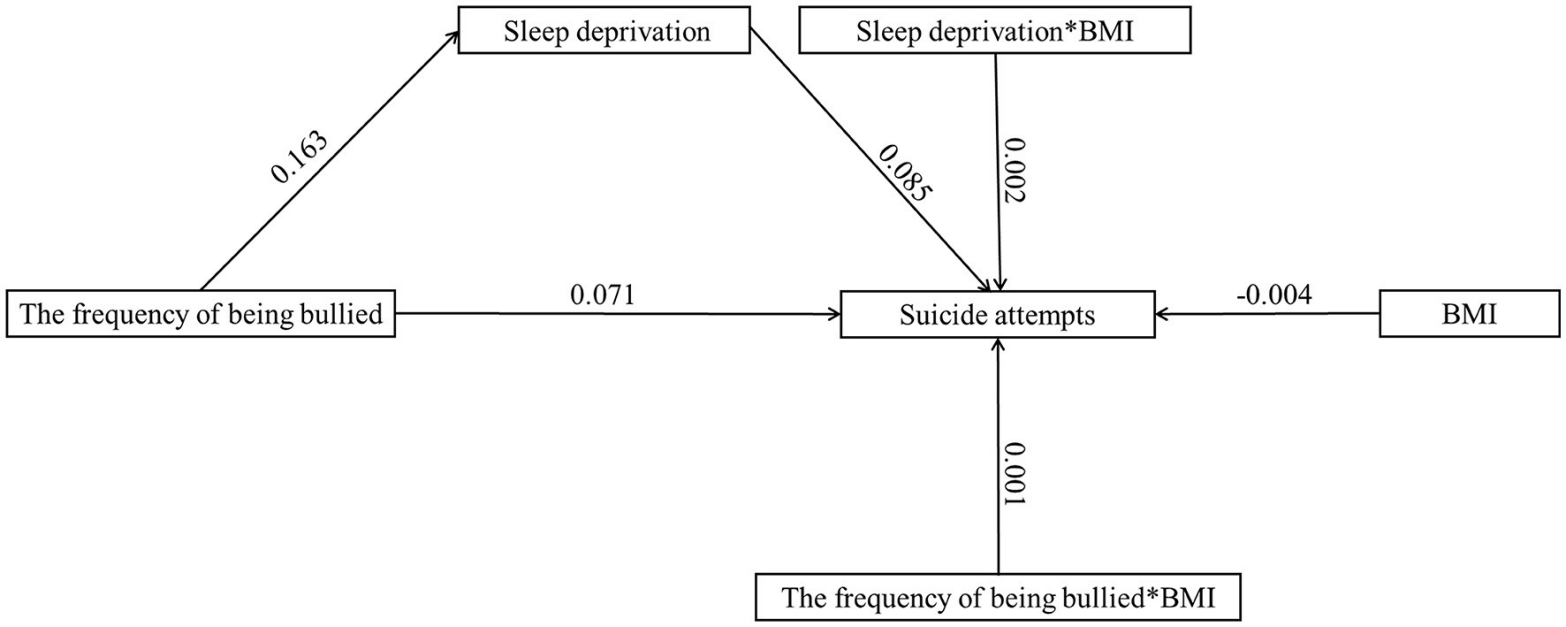
The moderated mediation model with the frequency of being bullied (*n* = 121,869).

**Figure 2:**
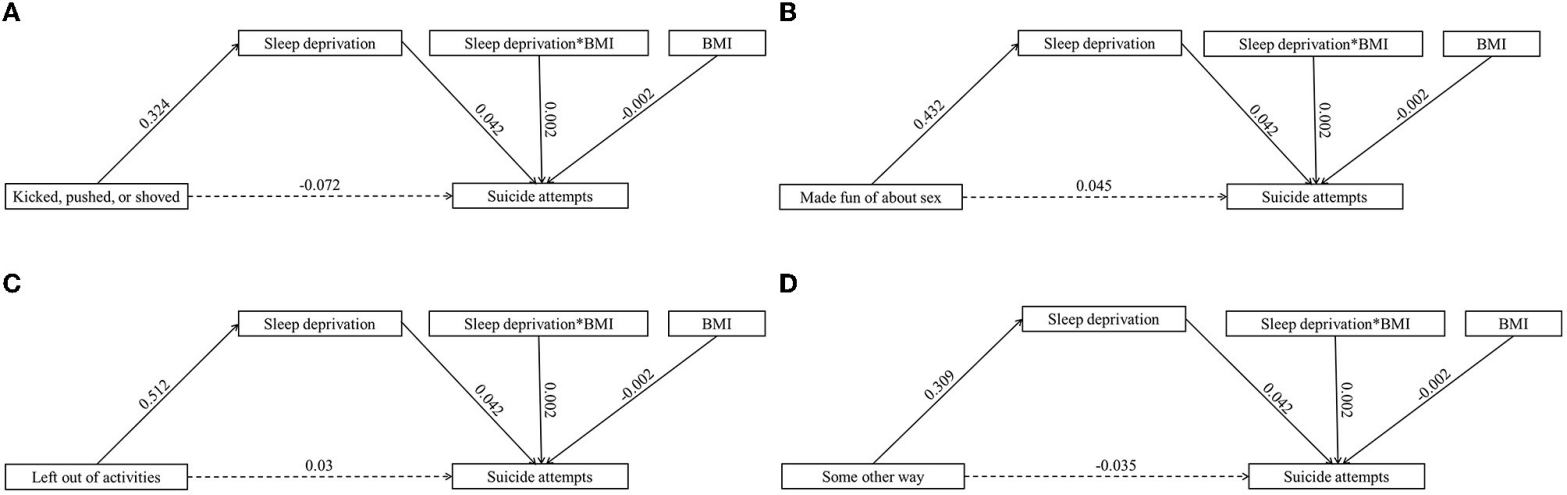
The moderated mediation model with different types of bullying (full mediating effects) (*n* = 121,869). **(A)** Kicked, pulsed, or showed. **(B)** Made fun of about sex. **(C)** Left out of activities. **(D)** Some other way.

**Figure 3:**
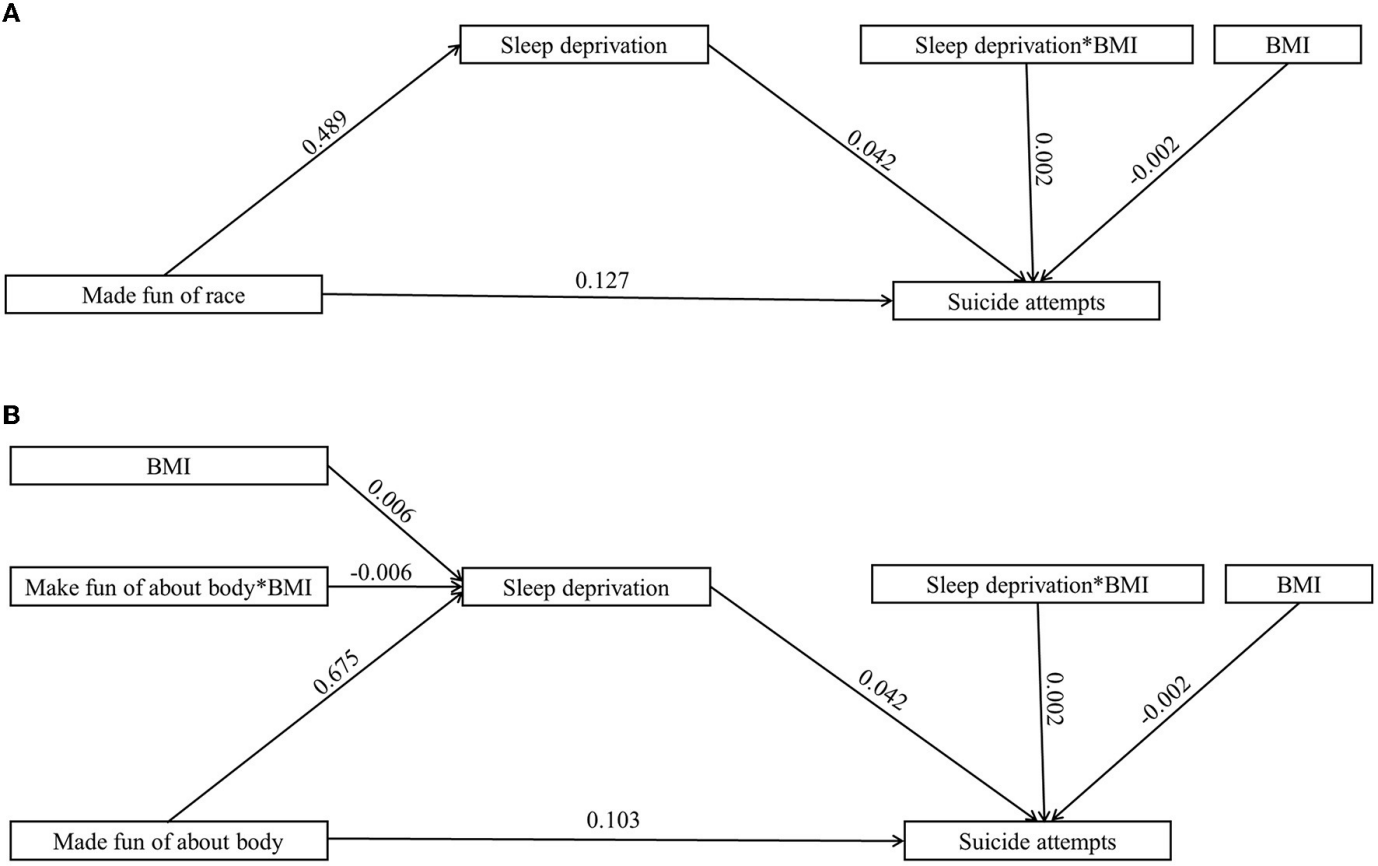
The moderated mediation model with different types of bullying (partial mediating effects) (*n* = 121,869). **(A)** Made fun of race. **(B)** Made fun of about body.

Table 3 illustrates the moderating effect results for BMI. Adding BMI to the model led to the following results: (a) frequency of being bullied (see Figure 1): the product of the frequency of being bullied and BMI had a significant predictive effect on suicide attempts (β = 0.001, *t* = 4.254, *p* < 0.001). According to simple slope analysis, bullying frequency was positively associated with suicide attempts among adolescents with low BMI (simple slope = 0.066, *t* = 33.800, *p* < 0.001) and high BMI (simple slope = 0.077, *t* = 41.125, *p* < 0.001). The product of sleep deprivation and BMI had a significant predictive effect on suicide attempts (β = −0.002, *t* = 6.213, *p* < 0.001). In simple slope analysis, it was found that sleep deprivation was positively related to suicide attempts among adolescents with low BMI (simple slope = 0.076, *t* = 35.893, *p* < 0.001) and high BMI (simple slope = 0.094, *t* =45.830, *p* < 0.001). These results indicate that as the frequency of being bullied and sleep deprivation increased, the suicide attempt of high-BMI adolescents increased more significantly. (b) Types of bullying (see Figures 2, 3): the product of being made fun of about one's body and BMI had a significant predictive effect on sleep deprivation (β = −0.006, *t* = −2.268, *p* < 0.05). Further simple slope analysis showed that being made fun of about one's body was positively associated with sleep deprivation among adolescents with low BMI (simple slope = 0.564, *t* = 28.952, *p* < 0.001) and high BMI (simple slope = 0.523, *t* = 32.850, *p* < 0.001). These results indicate that as being made fun of about one's body increased, the sleep deprivation of low-BMI adolescents increased more significantly. Moreover, the product of sleep deprivation and BMI had a significant predictive effect on suicide attempts (β = 0.002, *t* = 6.037, *p* < 0.001) for all types of bullying. According to a simple slope analysis, a positive association was found between sleep deprivation and suicide attempts among adolescents with low BMI (simple slope = 0.079, *t* = 39.964, *p* < 0.001) and high BMI (simple slope = 0.093, *t* =51.880, *p* < 0.001). These results indicate that as sleep deprivation increased, the suicide attempt of high-BMI adolescents increased more significantly.

**Table 3:** Moderating effect of BMI (*n* = 121,869).

	**Model 1**		**Model 2**	
	**Sleep deprivation**		**Suicide attempts**	
**Variable**	β	***t***	β	***t***
The frequency of being bullied	0.163	62.713^***^	0.071	51.619^***^
Sleep deprivation			0.085	56.885^***^
BMI			−0.004	9.515^***^
The frequency of being bullied^*^BMI			0.001	4.254^***^
Sleep deprivation^*^BMI			0.002	6.213^***^
*R* ^2^	0.031		0.057	
*F*	3,932.895		1,480.207	
Kicked, pushed, or shoved	0.324	3.18^**^	−0.072	−1.344
Made fun of race	0.489	4.746^***^	0.127	2.337^*^
Made fun of about sex	0.432	5.177^***^	0.045	1.014
Left out of activities	0.512	3.895^***^	0.03	0.436
Made fun of about body	0.675	10.656^***^	0.103	3.078^**^
Some other way	0.309	5.008^***^	−0.035	−1.077
Sleep deprivation			0.042	5.599^***^
BMI	0.006	7.692^***^	−0.002	−2.515^*^
Made fun of about body^*^BMI	−0.006	−2.268^*^		
Sleep deprivation^*^BMI			0.002	6.037^***^
*R* ^2^	0.038		0.051	
*F*	320.371		387.750	

* *p* < 0.05,

** *p* < 0.01,

*** *p* < 0.001.

## Discussion

Bullying is a serious social behavior problem; bullying has negative health consequences for victims and is a stressor leading to victims' suicidal behaviors (31, 36). Suicide is a major cause of death for adolescents and arouses widespread social concern. Considering that almost half of the world's youth (15–24 years) live in LMICs (58), research on suicide among these adolescents is essential. In this study of adolescents in 41 LMICs, we found a correlation between bullying victimization and suicide attempt; as the frequency of being bullied increases, the rate of suicide attempts by victims generally increases. Therefore, being bullied is positively associated with suicide attempts among adolescents, and this applies to different types of bullying, which is in line with Hypothesis 1. The frequency of being bullied and the type of bullying have different effects on adolescents' suicide attempts. In addition, sleep deprivation and body mass, as diathesis factors of suicide, also play a role in this process, which we will discuss concretely below.

### The impact of different types of bullying on suicide attempts

Through the analysis of the data, we found a correlation between bullying victimization and suicide attempts and observed that different types of bullying had varying degrees of impact on victims' suicide attempts. Bullying is a common adverse life event in adolescents, especially in LMIC, and a study recently conducted in southwest China showed that ~12.41% of adolescents had been victimized by bullying (56, 59, 60). Previous studies have shown that the risk for bullying and victimization are not equal across student groups; a number of studies have indicated that students with disabilities and students with obesity, as well as those belonging to ethnic or sexual minorities, are at greater risk for being victimized than their peers (23–26). Our results indicate that adolescents who were bullied because of religion have the highest probability of suicide attempts, followed by those who were “kicked, pushed, or shoved” and “made fun of race”. This finding is similar to the research findings reported by Koyanagi et al. (61) and may have a possible link between identity cognition (such as religion and race). Minorities may experience greater stigma and insufficient self-identification due to identity-based bullying (IBB). A study by Galán et al. showed that there was a correlation between IBB victims' poor mental and physical health, including non-suicidal self-injury and suicidal ideation (62). However, the specific mechanism is still unknown. The impact of physical bullying, such as being “kicked, pushed, or shoved”, on adolescents' suicide attempts may be derived from two aspects, i.e., mental and physical. The study by Thomas et al. showed that physical bullying was associated with high levels of psychological distress and reduced emotional wellbeing regardless of its frequency (63). However, victims of physical bullying were more likely to suffer from physical pain and injury, which may affect their physical function and health. Exposure to painful and provocative events could make adolescents more likely to engage in behaviors leading to suicide (64). The participants in this study were adolescents in LMICs, but the correlation between bullying victimization and suicide attempts was consistent in both low-income and high-income countries. A meta-analysis conducted by Holt et al. of 47 studies mainly conducted in high- and middle-income countries also showed that bullying victimization was significantly positively correlated with suicidal behavior (65). Few previous studies have explored the impact of types of bullying on suicide attempts by victims, which is also one of the research topics of our study. According to the stress-diathesis model, even under the same stressor, different individuals will have different responses because of their own susceptibility. Our research also confirms this theory. The regression analysis showed that bullying victimization exerts a significant indirect impact on suicide attempts through sleep deprivation. Body mass also moderates the effects of bullying victimization on suicide attempts.

### The mediating effect of sleep deprivation

Consistent with Hypothesis 2, we found that sleep deprivation is a crucial explanatory mechanism linking bullying victimization to suicide attempts among adolescents in 41 LMICs. Specifically, adolescents exposed to a higher frequency of being bullied were prone to have a higher risk of sleep deprivation, which in turn was related to suicide attempts. The results were also true for different types of bullying. This study supported previous studies showing a robust association between bullying victimization and sleep problems (i.e., difficulties falling or remaining asleep). The effects of sleep deprivation on children can be particularly detrimental, impacting their ability to learn, manage mood and anxiety, develop relationships, avoid accidents, and stay physically healthy (42). A number of recent studies have identified sleep deprivation as a modifiable, independent suicide risk factor (44). Therefore, sleep deprivation is not only ascribed to bullying victimization but also acts as an internal antecedent of suicide attempts. These findings extend beyond existing studies by revealing how bullying victimization is associated with adolescent suicide attempts and suggest that the frequency of being bullied and various types of bullying behavior may be closely related to adolescents' quality of sleep.

Aside from the overall mediation result, each of the individual links deserves mention. Regarding the first part of the mediation process (i.e., bullying victimization → sleep deprivation), our findings suggest that the frequency of being bullied is positively related to sleep deprivation. This finding is congruent with previous research that suggested that bully victimization and loneliness were consistently and positively linked to SLOW, with greater odds of sleep deprivation seen among students with severe SLOW who were bullied for 3 days or more (46). Victims of bullying have been found to suffer from anxiety, posttraumatic stress, depression, and suicidal ideation, as well as somatic conditions such as headaches and stomachaches. At the same time, sleep has been associated with these negative psychosocial outcomes commonly seen among both bullies and victims (47). With the occurrence of bullying, the victim develops emotional distress and psychological stress, which can lead to sleep deprivation. In the second phase of the mediation process (i.e., sleep deprivation → suicide attempt), we found that sleep deprivation was positively associated with suicide attempts. Sleep deprivation was associated with suicide attempts, which is supported by robust evidence. For example, a review by Zullo et al. showed that sleep deprivation and eveningness uniquely contributed to poor daytime functioning and mood-related outcomes, while the coexistence of these two conditions could confer a greater risk in adolescents. Beyond that, studies have shown that improved sleep might reduce suicide risk (45).

The results could also apply to different types of bullying. Similar intermediate relationships existed between different types of bullying and sleep deprivation. Previous studies have shown that a similar pattern was found across all types of bullying, with more sleep disturbances for victims and bully victims (47). In this study, being made fun of about one's body is the bullying style with the most positive impact on sleep deprivation. The second is being left out of activities. Being kicked, pushed, or shoved has the least effect on sleep deprivation. Therefore, we can infer from this study that verbal bullying has the greatest impact on sleep deprivation, followed by social bullying, and that physical bullying has the least impact. The results are consistent with previous studies. Verbal bullying was found to be easier to implement and to be the dominant type of bullying among adolescents, reaching a prevalence of 53.2% (66). Both victim-only and bully victims involved in verbal bullying also reported having more bedtime fears than bully only youth, while bully victims involved in physical bullying reported more bedtime fears than bully only youth. Sleep deprivation was the highest among bully victims involved in verbal bullying, while sleep deprivation was higher among victim-only youth involved in social bullying (47). These types of bullying had a positive effect on sleep deprivation, which could lead to suicide attempts.

Notably, this study demonstrated that sleep deprivation only partially mediates the relation between the frequency of being bullied and suicide attempts. This may be because personal definitions of bullying are different, leading to deviations in the frequency of being bullied. The mediating effect of sleep deprivation on the relationship between bullying type and suicide attempt was not only fully mediated but also partially mediated. Being “kicked, pushed, or shoved,” “left out of activities,” and “made fun of about one's sex” play a full mediating role, while being “made fun of about one's race” and “made fun of about one's body” played a partial mediating role. In spite of this, sleep deprivation is an important linking mechanism that deserves special attention. Notably, being a victim of school bullying was found to be significantly associated with reports of suicidal ideation and attempts (15). In summary, bullying victimization can be considered a significant factor in adolescent suicide in LMICs. Sleep deprivation plays crucial direct and indirect roles in suicide attempts, the extent and mechanisms of which urgently require investigation.

### Body mass as a moderator

Consistent with the results of hypothesis 3, the results of this research reveal that body mass moderates the relationship between bullying victimization and suicide attempts. The effects are stronger among adolescents with a high BMI than among those with a low BMI. The results of data analysis show that high BMI alone is a protective factor against suicide attempts. People who are underweight are more likely to attempt suicide, which is consistent with most previous studies (49, 67). In addition, body mass plays a moderating role in the path of bullying victimization to suicide attempts. Among adolescents with different BMIs, the degree of influence of being bullied on suicide attempts differs. Adolescents with high BMI are more likely to attempt suicide after being bullied. The current understanding of the mechanism of this regulation is still unclear, but it may be related to the fact that adolescents with higher BMI are more likely to be bullied (25, 51). Moreover, the product of sleep deprivation and body mass has a significant predictive effect on suicide attempts. This finding suggests that body mass also moderates the effect of sleep deprivation on suicide attempts. The data analysis showed that as the frequency of being bullied and sleep deprivation increased, the suicide attempt of high-BMI adolescents increased more significantly. Based on a series of previous studies, we found that there is a vicious cycle between obesity and lack of sleep (48, 54). Obese people are prone to lack of sleep, and poor sleep can also exacerbate the occurrence of obesity. At the same time, sleep deprivation can also act as an intermediary factor between bullying victimization and suicide attempts, leading victims to attempt suicide. Therefore, it is not difficult to infer that BMI can further increase the probability of suicide by bullying victims through its positive effects on sleep deprivation. In addition, previous studies have found that obese people had difficulty regulating their emotions and were less aware of their interoceptive senses (68). This means that obese people may have more difficulty dealing with the negative emotions brought about by being bullied and are more likely to have suicidal impulses.

In terms of the moderating role of body mass in the influence of different types of bullying on suicide attempts, the product of being made fun of about one's body and body mass has a significant predictive effect on sleep deprivation. When adolescents with low BMI were made fun of by their bodies, they were more likely to suffer from sleep deprivation. This finding is very important because previous studies have shown that obese people were more prone to sleep deprivation. On the one hand, this sleep deprivation may be related to sleep interruption caused by diseases such as obstructive sleep apnea, gastroesophageal reflux disease, and asthma, which are diseases that obese people easily suffer from. On the other hand, sleep deprivation may also be related to the close relationship between obesity and depression (69). Regarding why adolescents with low BMI are more prone to sleep deprivation after being made fun of about their bodies, there is no appropriate explanation. This may be because adolescents with low BMI are thinner and more vulnerable to physical harm after being bullied, which in turn leads to an aggravation of sleep deprivation. For these types of bullying (being kicked, pushed, or shoved; being made fun of about one's race; being made fun because of religion; being made fun of about one's sex; being left out of activities; being made fun of about one's body; being bullied some other way), body mass moderates the effect of sleep deprivation on suicide attempts. As sleep deprivation increases, the suicide attempt of high-BMI adolescents increases more significantly, which is consistent with the moderating path of the frequency of being bullied (48, 54, 68). Coupled with the moderating effect of body mass on suicide attempts, body mass moderates the complete path of bullying by being made fun of about one's body through sleep deprivation, leading to suicide attempts. As body mass is closely related to body image, its moderating role in this path is reasonable. For other types of bullying, body mass does not moderate the relationship between being bullied and sleep deprivation. The specific mechanism is still unclear and needs further study.

Based on the above, we believe that bullying victimization is one of the stress components in the stress-diathesis model of suicide in adolescents. Under the stress of being bullied, different individuals respond differently to the stressor due to their own diathesis factors, and diathesis-related biomarkers or characteristics can help indicate suicide risk. Our study found that sleep deprivation plays a mediating role in the pathway of bullying victimization to suicide attempts in adolescents, and bullying victimization leads to sleep deprivation, which further induces the emergence of suicide attempts, while body mass plays a moderating role in this model, teenagers with BMI values not within the normal range are more likely to attempt suicide after being bullied. Our study also further verified the feasibility of the stress-diathesis theory in suicidal behavior and clarified the interaction between stress and diathesis components in the stress-diathesis model of suicide.

### Limitations and implications

There are a few limitations to our study that can be addressed through future research. First, although the model in our study is theoretically grounded and empirically supported, due to the shortcomings of cross-sectional designs, causality cannot be determined. There is a need for further longitudinal studies or intervention experiments to better examine the validity of this model. Additionally, the questionnaire used in the survey might lead to recall bias because it was self-reported. Third, the results of the survey may be biased by social desirability and different cultural factors across countries. Although the survey was anonymous, differences in sociocultural factors among countries can lead to different types of bullying, which might affect self-reporting about the frequency of being bullied and cause further potential bias. Finally, this survey did not measure adolescents' mental illness (depression, bipolar affective disorders, trauma, etc.) or previous psychological distress, which made them more vulnerable to the extent to which bullying victimization could easily trigger their suicide attempt. We suggest that further focused research is necessary to further understand the risk as children progress through adolescence, which is crucial for clinical management and disease prevention (70, 71).

Despite these limitations, our study has several strengths. First, the sample is large (126,763 young adolescents aged 12–17 years) and covers 41 LMICs in the five WHO regions; thus, it has good applicability and extension. In addition, this study has important theoretical and practical implications. Overall, this study provides valuable insight into the relationship between bullying victimization and suicide attempts, showing the mediating effects of sleep deprivation and the moderating role of body mass and offering a theoretical basis for creating a healthy school environment and promoting mental health among adolescents. In terms of practicality, our research results can provide a reference for the prevention of adolescent suicide attempts to reduce the incidence of teenage suicide, promoting the development of adolescents' physical health.

## Conclusion

Using a sample of adolescents in 41 LMICs, this study takes an important step in examining the mechanism by which bullying victimization is associated with suicide attempts. Bullying victimization has a positive effect on suicide attempts, which is mediated by sleep deprivation and moderated by body mass. This study provides insight into how bullying victimization impacts adolescents' suicide attempts. The findings can serve as theoretical bases for promoting adolescents' mental health and are conducive to identifying adolescents at a high risk of suicide, suggesting that more attention should be given to bullied adolescents, especially their sleep quality and body mass. Based on this study, we can design effective intervention measures to reduce the suicide possibility of bullied adolescents and improve the current situation of adolescent suicide in LMICs, such as improving sleep quality and maintaining a healthy body mass.

## Data availability statement

Publicly available datasets were analyzed in this study. This data can be found here: Global School-based Student Health Survey (GSHS) data presented on the websites of the WHO (http://www.who.int/ncds/surveillance/gshs/en/) and Centers for Disease Control and Prevention (CDC).

## Ethics statement

In each participating country, the GSHS administration is approved by the Ministry of Education, a Health Research Ethics Committee, or both. Verbal or written consent is obtained from all the participants and their guardians in each country. This study does not require the approval of ethics or institutional review boards because the analyses are based on publicly available data.

## Author contributions

WB, YQ, and WF: visualization, formal analysis, writing (original draft), and writing (review and editing). ST: writing (original draft). YG and SW: writing (review and editing). C-WP: conceptualization, writing (review and editing), and validation. C-HZ: conceptualization, visualization, formal analysis, writing (original draft), writing (review and editing), and validation. TZ: conceptualization, visualization, formal analysis, writing (original draft), writing (review and editing), validation, and funding acquisition. All authors read and approved the final manuscript.
